# A combinatorial MRI sequence-based radiomics model for preoperative prediction of microsatellite instability status in rectal cancer

**DOI:** 10.1038/s41598-024-62584-0

**Published:** 2024-05-23

**Authors:** Xiaowei Xing, Dongxue Li, Jiaxuan Peng, Zhenyu Shu, Yang Zhang, Qiaowei Song

**Affiliations:** 1 Cancer Center, Department of Radiology, Zhejiang Provincial People′s Hospital (Affiliated People′s Hospital), Hangzhou Medical College, Hangzhou, Zhejiang China; 2 Jinzhou Medical University, Jinzhou, Liaoning Province China

**Keywords:** Rectal cancer, Machine learning, Microsatellite instability, Magnetic resonance imaging, Radiomics, Cancer, Cancer imaging

## Abstract

This study aimed to develop an optimal radiomics model for preoperatively predicting microsatellite instability (MSI) in patients with rectal cancer (RC) based on multiparametric magnetic resonance imaging. The retrospective study included 308 RC patients who did not receive preoperative antitumor therapy, among whom 51 had MSI. Radiomics features were extracted and dimensionally reduced from T2-weighted imaging (T2WI), T1-weighted imaging (T1WI), diffusion-weighted imaging (DWI), and T1-weighted contrast enhanced (T1CE) images for each patient, and the features of each sequence were combined. Multifactor logistic regression was used to screen the optimal feature set for each combination. Different machine learning methods were applied to construct predictive MSI status models. Relative standard deviation values were determined to evaluate model performance and select the optimal model. Receiver operating characteristic (ROC) curve, calibration curve, and decision curve analyses were performed to evaluate model performance. The model constructed using the k-nearest neighbor (KNN) method combined with T2WI and T1CE images performed best. The area under the curve values for prediction of MSI with this model were 0.849 (0.804–0.887), with a sensitivity of 0.784 and specificity of 0.805. The Delong test showed no significant difference in diagnostic efficacy between the KNN-derived model and the traditional logistic regression model constructed using T1WI + DWI + T1CE and T2WI + T1WI + DWI + T1CE data (*P* > 0.05) and the diagnostic efficiency of the KNN-derived model was slightly better than that of the traditional model. From ROC curve analysis, the KNN-derived model significantly distinguished patients at low- and high-risk of MSI with the optimal threshold of 0.2, supporting the clinical applicability of the model. The model constructed using the KNN method can be applied to noninvasively predict MSI status in RC patients before surgery based on radiomics features from T2WI and T1CE images. Thus, this method may provide a convenient and practical tool for formulating treatment strategies and optimizing individual clinical decision-making for patients with RC.

## Introduction

The incidence and mortality rates of rectal cancer (RC) have increased significantly in recent years, with RC now having the second and third highest incidence and mortality rates, respectively, among all cancer types^1^. The two main molecular pathways for the occurrence of RC are chromosomal instability (CIN) and microsatellite instability (MSI)^2^. MSI results from a deoxyribonucleic acid (DNA) mismatch repair defect (dMMR) and is an essential characteristic used for early screening and diagnosis of RC. The detection of MSI represents a vital predictive tool for evaluating the prognosis of patients and predicting the response to adjuvant therapies and immunotherapies, thereby guiding the selection of such therapies for individual patients. Research has shown that RC patients with MSI has unique biological behaviors and distinct responses to treatment, and may be resistant to 5-fluorouracil (5-FU)–based chemotherapy and more likely to benefit from immunotherapy^3^. Since 2016, several major oncology groups, including the National Comprehensive Cancer Network (NCCN), European Society for Medical Oncology (ESMO), and Chinese Society of Cancer Clinical Oncology (CSCO), have all recommended MSI evaluation for RC patients^4,5^. Traditional MSI testing is mainly based on colonoscopy and invasive biopsy for evaluation; however, the DNA extracted from the sample obtained via colon biopsy may not meet the minimum quality criteria from genetic analysis. Additionally, because of the high heterogeneity of colorectal cancer, the results of MSI detection may vary depending on insufficient samples or sampling techniques^6^. In addition, the risks and complications of invasive biopsy limit its application in the real-time monitoring of disease progression and biological behaviors. Therefore, there is an urgent need for a non-invasive and accurate method to determine the preoperative MSI status of RC cases.

Radiomics has been applied to develop valuable methods for tumor detection and diagnosis as well as the prediction of prognosis and treatment response^7,8^. Previous studies have investigated whether magnetic resonance imaging (MRI) radiomics data can be used to predict MSI status in RC patients^9–11^, and their results have shown the potential of MRI radiomics approaches for predicting MSI status in RC. However, these early studies used a single sequence to study MSI status and did not explore which combinations of MRI sequences are most suitable for predicting MSI status. On the other hand, predictive models are essential in radiomics approaches, and accurate and reliable models are the key to improving the decision-making process in clinical practice. Extensive research has demonstrated the value of machine learning algorithms in the development of predictive models for radiomics approaches. We predicted that combining machine learning with multi sequence MRI data can improve the accuracy of radiomics in predicting MSI.

Therefore, the first aim of this study was to identify the optimal sequence combination that can predict the MSI status of RC patients. Using the optimal sequence combination identified in combination with machine learning, the second aim of this study was to construct a prediction model for the preoperative identification of MSI status. The developed model was then validated to demonstrate its potential for supporting personalized precision medicine in RC treatment.

## Methods

### Patient data

**Figure 1:**
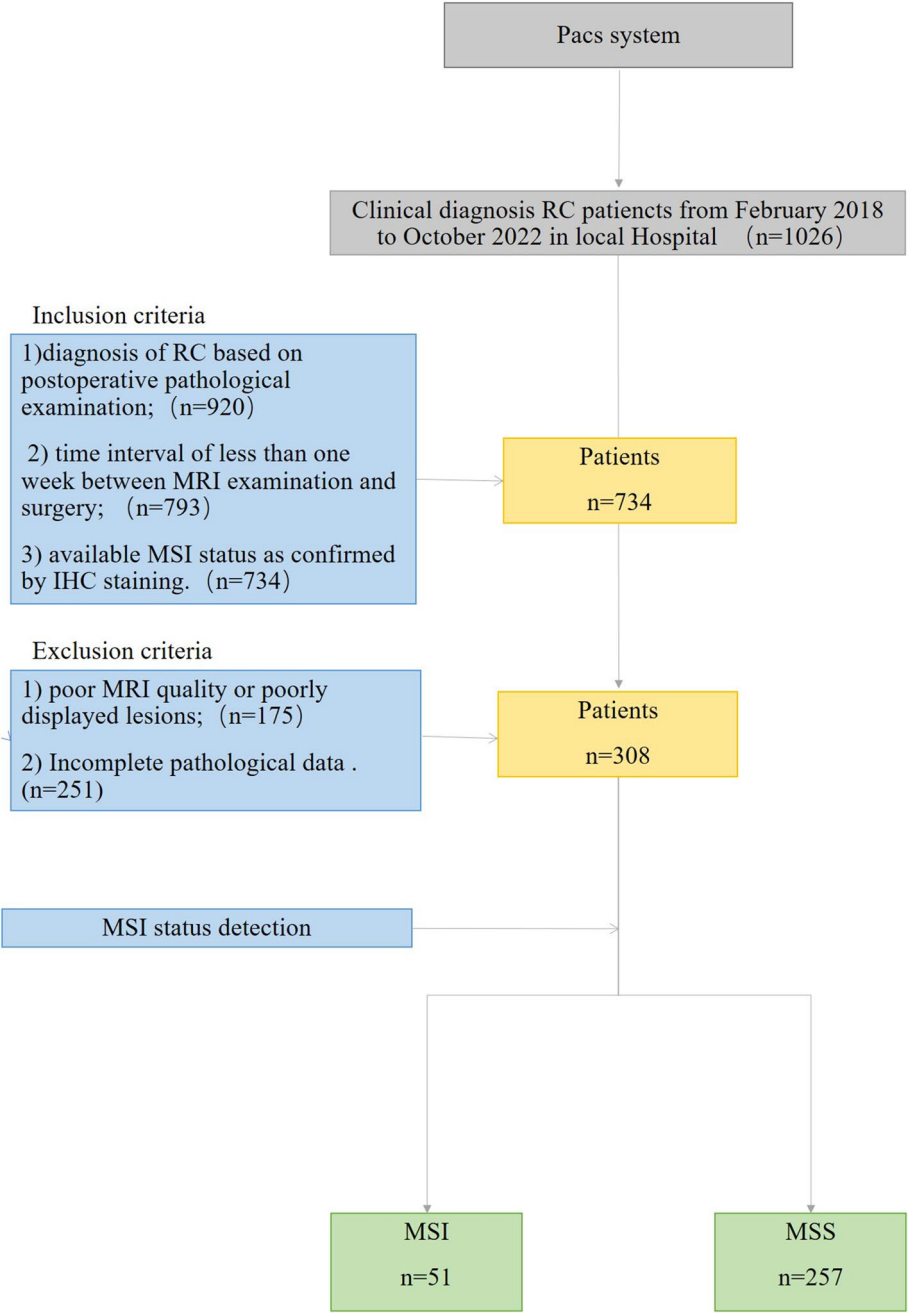
Flowchart of patient inclusion.

This retrospective study was approved by the Ethics Committee of Zhejiang Provincial People's Hospital (NO.2021QT256), without a requirement of informed consent. This study handled all patient data confidentially and complied with the Declaration of Helsinki. A total of 1026 patients who received a clinical diagnosis of RC between February 2018 and October 2022 and had images available in the hospital’s archiving and communication system were reviewed. Patients were then included in this retrospective study based on the following inclusion and exclusion criteria. The inclusion criteria were: (1) diagnosis of RC based on postoperative pathological examination; (2) time interval of < 1 week between MRI examination and surgery; and (3) available MSI status as confirmed by immunohistochemistry staining. The exclusion criteria were: (1) poor MRI quality or poorly displayed lesions; and (2) incomplete pathological data. Finally, 308 patients were enrolled. The inclusion process for the research cases is presented in Fig. 1.

### Clinicopathological and laboratory variables

The following clinicopathological characteristics of RC patients were recorded: age, gender, T-stage and N-stage based on MRI, distance from the end of the convex edge of the tumor to the edge of the anus (DIS), circumferential resection margin on MRI (CRM) status, and extramural venous invasion (EMVI). The specific definitions of these clinical features are provided in the supplementary materials. Two radiologists with extensive RC diagnostic experience (Radiologist A and Radiologist B, with 5 and 16 years of abdominal imaging experience, respectively) independently reviewed the above tumor imaging features in a double-blind setting. The final results for all quantitative data were the average of the measured values from the two radiologists, and the final results for all qualitative data were determined through consultation when the two radiologists had different opinions. Laboratory analyses included measurement of carcinoembryonic antigen (CEA) and carbohydrate antigen 19–9 (CA19-9) levels. The levels of these markers were considered abnormal at threshold values of > 5.0 ng/mL and > 37.0 U/mL for CEA and CA19-9, respectively.

### MSI status detection

The MSI status of patients was determined by immunohistochemical staining for the following mismatch repair (MMR) proteins: mutL homolog 1 (MLH1), mutS homolog 2 (MSH2), mutS homolog 6 (MSH6), and post-meiotic segregation increased 2 (PMS2). Staining for these proteins was performed using the standard streptavidin–biotin–peroxidase procedure. The MSI status of a case was considered positive if staining for any one (or more) of the four MMR proteins was negative. The MSI status of a case was considered negative if staining for all four MMR proteins was positive; these cases were considered to have microsatellite stability (MSS)^12^.

### MRI imaging preprocessing process

mp-MRI examinations for all patients were performed using a 3.0T MRI scanner (Skyra; Siemens Healthineers). The protocols and detailed parameters use for mp-MRI are described in detail in the Supplementary Materials and Table S1. All layers of the patients’ MRI images were segmented (version 3.6.0; www.itksnap. org) using ITK-SNAP software, and data from air, feces, and fat around the colon were eliminated to the greatest extent possible. In this study, T2-weighted imaging (T2WI) was conducted perpendicular to the rectum axis as the template for rigid registration of all sequences to ensure that the four sequences contained the same resolution, spacing, and origin. The standardized T2WI images were imported into the ITK Software to segment the entire rectal tumor layer by layer to determine the volume of interest (VOI). Finally, depending on the registration of sequences, T1-weighted imaging (T1WI), diffusion-weighted imaging (DWI), and T1-weighted contrast enhanced imaging (T1CE) could share the same VOI from T2WI for extraction.

To confirm the reproducibility of the VOI and the robustness of the imaging features, tumor images were segmented by Radiologists A and B, separately. The radiomics features were extracted from the segmented lesions and divided into two feature sets (feature set A and feature set B). Spearman’s rank correlation test was used to calculate the correlation coefficient (CC) for each feature in sets A and B. Features with CC > 0.8 were considered robust.

### Extraction of radiomic features

Before extraction of radiomics features from MRI images, the images were preprocessed using three steps. First, each sequence of an image was resampled at a 1 × 1 × 1 mm^3^ resolution by linear interpolation to reduce the impact of variation in layer thicknesses. Second, the gray level of the image was discretized and normalized to 25 orders. Third, during image digitization, the Laplacian of Gaussian (LoG) and wavelet methods were applied to eliminate mixed noise and produce low- and high-frequency features. In total, 996 features within seven categories were extracted from original MRI images obtained by T2WI, T1WI, DWI, and T1CE as well as wavelet-filtered images and LoG-processed images using PyRadiomics software^13^. More detailed explanations of the feature extraction methods are presented in the Supplementary Materials.

Each radiomics feature was standardized with a z-score, and all missing data in the data queues were replaced with median values. All cases were classified according to MSI status as having either MSS or MSI. For these two categories, data from T2WI, T1WI, DWI and T1CE sequences were screened for relevant features. First, univariate analysis was used to identify the characteristics that differed between the MSS and MSI groups with a *P* < 0.05. Second, the least absolute shrinkage and selection operator (LASSO) method was used to identify features closely related to MSI status, and the significance of features was determined by observing the coefficients of predictive variables. Finally, the gradient boosting decision tree (GBDT) algorithm was applied to further retain features related to MSI status. The features extracted from each sequence were filtered according to the above dimensionality reduction steps to select the feature set that is most relevant to clinical results. In addition, different combinations of the most relevant features in four sequences were analyzed, and 11 combined feature sets were obtained by combining different sequences. To prevent overfitting, we conducted multifactor logistic regression for each combined feature set based on the stepwise searching approach and the stopping rule of Akaike’s information criteria to screen the optimal features and construct a traditional logistic regression model.

### Establishment of optimal radiomics model based on machine learning

**Figure 2:**
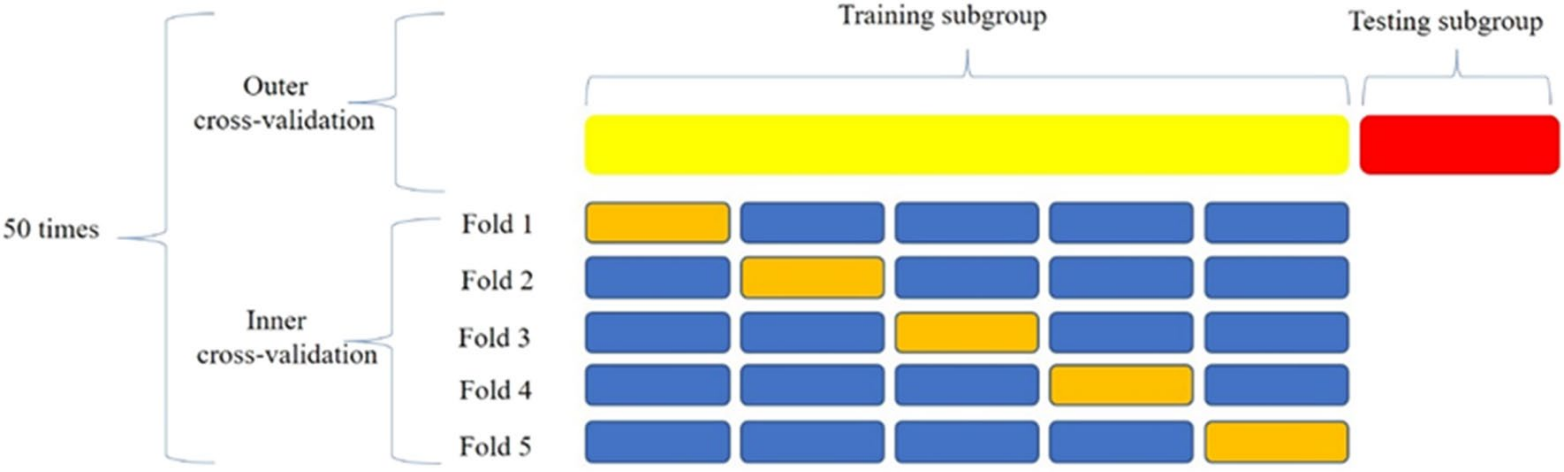
Cross-validation procedure of machine learning algorithm in the training cohort.

For each optimal feature set, we applied five machine learning algorithms to construct radiomics models, separately. The five machine learning algorithms were support vector machine (SVM), Bayes, k-nearest neighbor (KNN), decision tree, and random forest. To select the optimal machine learning algorithm for each feature set, we used the cross validation process for all data. The cross validation process of the machine learning algorithm includes two nested loops: one outer loop has a repeated hierarchical random segmentation training queue, in which 50 repetitions are used to evaluate the classification performance, and divide all cases into training subgroups and testing subgroups at a 7:3 ratio each time. The other inner loop has fivefold cross validation, which is used to optimize the hyper parameters of the algorithm, including the C-value of SVM, the n-neighbors of KNN, the max_depth of the decision tree, and n-estimators of random forest. One model is created for each segmentation, resulting in 50 models. Finally, the model with the highest accuracy in the test subgroup was selected for this study. See Fig. 2 for details. Then, we determined relative standard deviation (RSD) values to quantify the performance and stability of the five machine learning algorithms for each feature set. The machine learning algorithm with the lowest RSD value was selected as the best algorithm to construct the radiomics model. The models produced a radiomics (RAD) score for each patient, which reflected the likelihood of MSI. The RSD is the absolute value of the coefficient of variation and is often expressed as a percentage according to Eq. (1).

1 $$RSD = \frac{{\sigma_{AUC} }}{{\mu_{AUC} }} \times 100$$

where σ_AUC_ and µ_AUC_ are the standard deviation and mean of the 50 AUC values, respectively. It should be noted that higher stability corresponds to a lower RSD value.

### Evaluation and validation of the radiomics model

**Figure 3:**
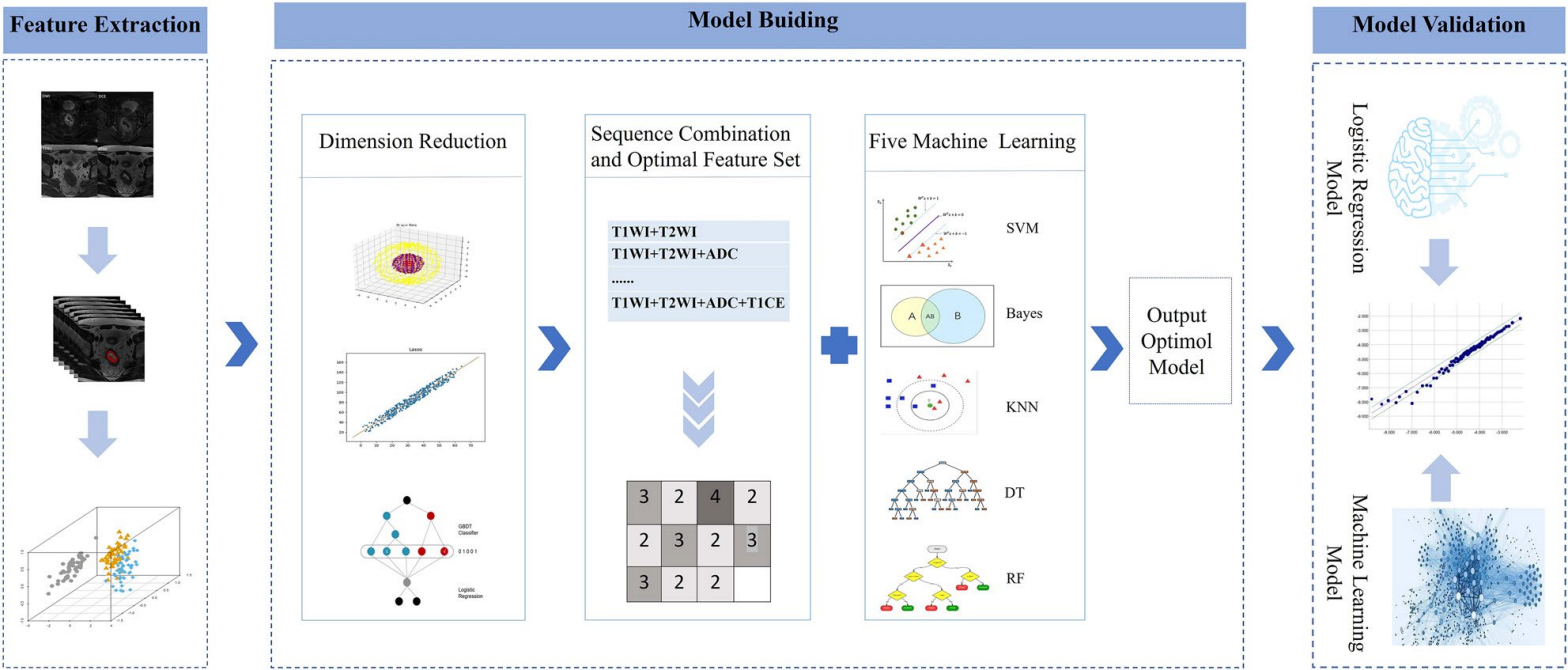
Research flow chart.

The predictive accuracy of the radiomics models was evaluated by calculating the area under the curve (AUC), 95% confidence interval (CI), accuracy, sensitivity, and specificity of the model for the entire cohort from receiver operating characteristic (ROC) curves. The Delong test was used to compare AUC values between the optimal radiomics model and traditional models. Calibration curves were generated to determine the consistency between the predicted MSI status and actual probability of the MSI status. Decision curve analysis (DCA) was applied to quantify the net benefits of model use under different threshold probabilities to assess the clinical utility of the radiomics model. Finally, the Youden index corresponding to the optimal cutoff value from ROC curve analysis was applied to divide the patient cohort into low- and high- probability groups for MSI. The results from the prediction model were compared with pathological results to evaluate the predictive accuracy of the model. A flow chart outlining model development is shown in Fig. 3.

### Statistical analysis

The R statistical software (version 3.6.3; http://www.Rproject.org) was used for constructing machine learning models, and the SPSS (version 24.0) and MedCalc (version 11.2) programs were used for statistical analyses in this study. The Kolmogorov‒Smirnov test was used to evaluate the normality of variable distribution. If the distribution of continuous variables was normal, an independent-samples t test was used to evaluate group differences. For continuous data without a normal distribution, the Mann–Whitney U test was used. For categorical data, the chi-square test or Fisher’s exact test was used to evaluate differences between groups. Differences were considered statistically significant if *P* < 0.05.

### Ethics approval and consent to participate

This retrospective study was approved by the Medical Ethics Committee of Zhejiang Provincial People’s Hospital (No. 2021QT256) and in conformity to the Declaration of Helsinki. The requirement of informed consent was waived for this retrospective study by the Medical Ethics Committee of Zhejiang Provincial People’s Hospital (No. 2021QT256) based on the retrospective nature of the study.

## Results

### Basic and clinical characteristics of RC patients according to MSI status

The average age of all patients was 64.22 years (range, 45.8–72.9 years), with males accounting for 70.13% of the total study population. No significant differences in age, gender, and CEA level were detected between the MSS and MSI groups (*P* > 0.05). The MSS and MSI groups also showed no significant differences in the imaging markers DIS, CRM, EMVI, ACI, lymph node metastasis, or tumor T stage (all *P* > 0.05; Table 1).

**Table 1:** Basic and clinical characteristics of patients in the MSS and MSI groups.

Characteristics	All patients (*n* = 308)	MSS group (*n* = 257)	MSI group (*n* = 51)	*P*-value
Age, years (mean, SD)	64.22 (10.2)	64.29 (10.2)	63.9 (10.3)	0.807
Male gender (n, %)	216 (70.13)	184 (71.6)	32 (62.75)	0.207
CEA level (abnormal, %)	124 (40.26)	104 (40.47)	20 (39.22)	0.868
DIS (cm, SD)	8.04 (3.75)	7.99 (3.68)	8.3 (4.12)	0.59
CRM status (positive, %)	72 (23.38)	58 (22.57)	14 (27.45)	0.452
EMVI status (positive, %)	75 (24.35)	58 (22.57)	17 (33.33)	0.102
ACI status (positive, %)	16 (5.19)	13 (5.06)	3 (5.88)	0.918
Lymph node metastasis (positive, %)	195 (63.31)	158 (61.48)	37 (72.55)	0.134
Tumor stage (n, %)				
T_1-2_	84 (27.27)	74 (28.79)	10 (19.61)	0.178
T_3-4_	224 (72.73)	183 (71.21)	41 (80.39)	

Data are presented as count (percentage) or mean (standard deviation).

MSS, microsatellite stability; MSI, microsatellite instability; CEA, carcinoembryonic antigen; DIS, distance from the end of the convex edge of the tumor to the edge of the anus; CRM, circumferential resection margin; EMVI, extramural vascular invasion; ACI, anal canal invasion.

### Selection of optimal combinations of sequence features and machine learning methods

**Figure 4:**
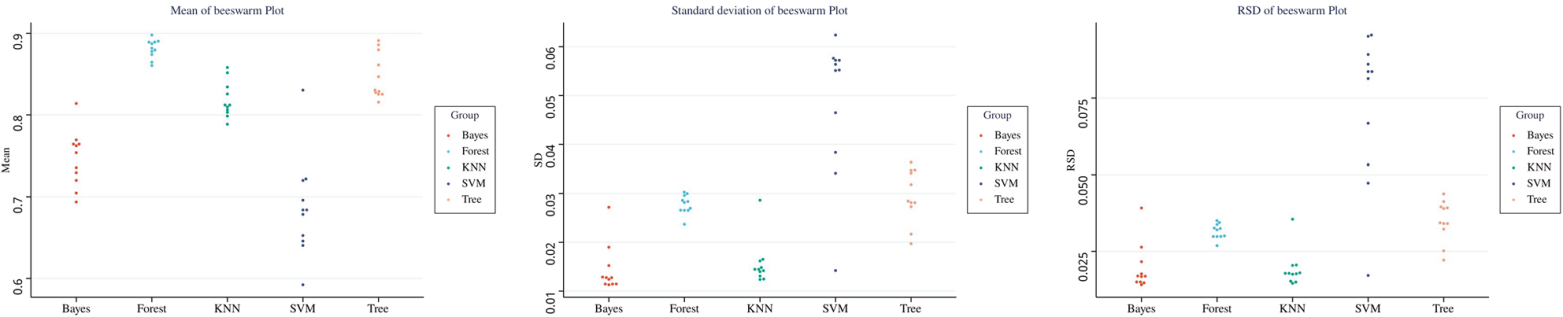
Average, standard deviation, and RSD values for the diagnostic effectiveness of radiomics models obtained using five machine learning algorithms. RSD is the ratio of the standard deviation to the mean of AUC values obtained from 50 machine learning model building cycles.

**Figure 5:**
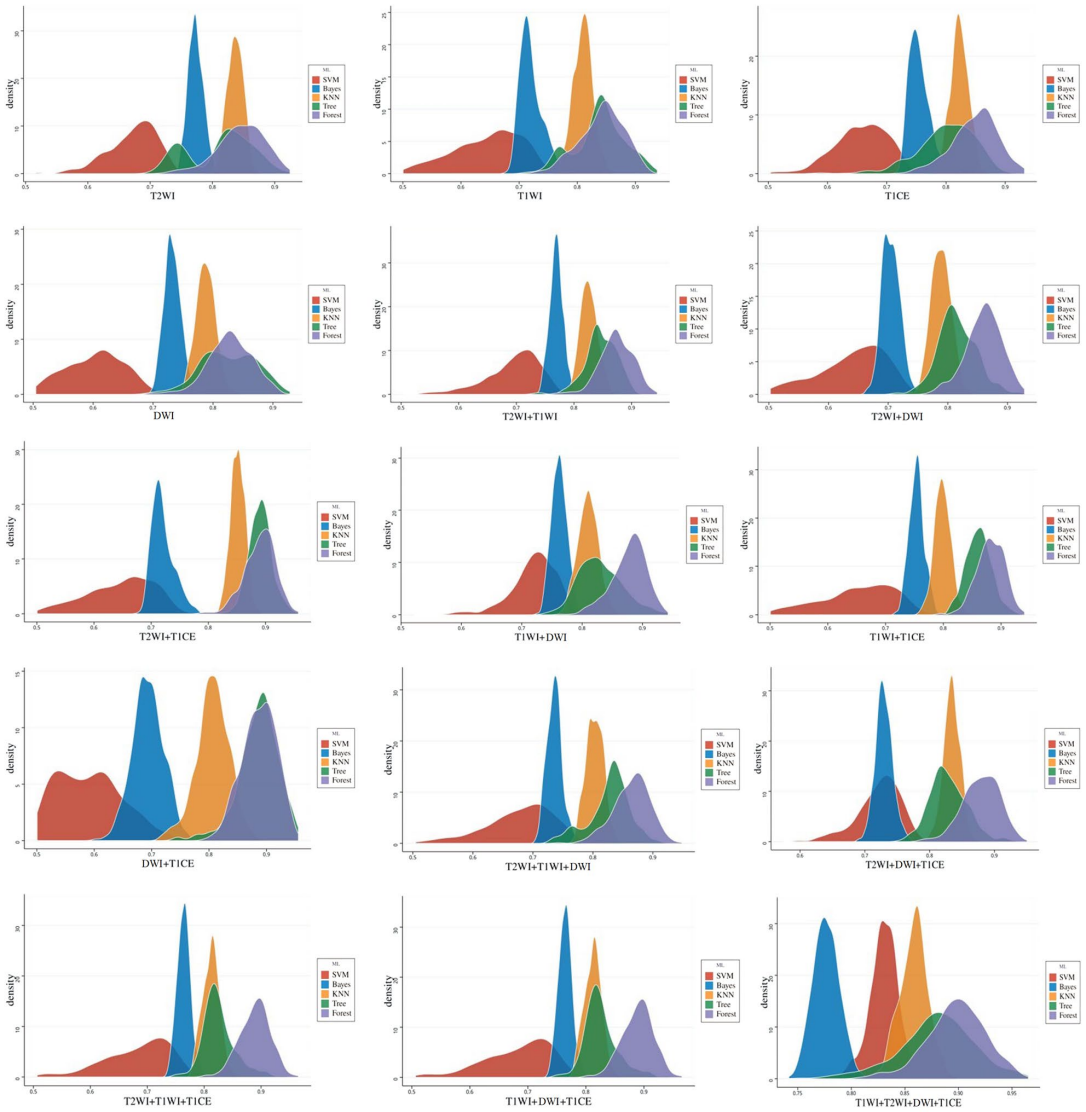
AUC density maps for radiomics models constructed using different machine learning methods for different combinations of sequence features.

After the dimensionality reduction of 3984 radiological features from four sequences, 47 features were obtained, including 11 features on T2WI, 11 on T1WI, 10 on DWI, and 15 on T1CE. We then analyzed combination of these features through multiple-factor logistic regression and obtained 11 optimal feature sets. Detailed descriptions of these optimal feature sets is provided in the Supplementary Materials. Based on the optimal feature sets, a radiomics model was constructed using machine learning, and RSD values were calculated (Fig. 4). The model constructed using the KNN method combined with features on T2WI and T1CE sequences had the lowest RSD value of 0.014523 and a medium average AUC value of 0.852, whereas the model constructed using the random forest method combined with four sequence features had a larger RSD value of 0.030046 and the highest average AUC value of 0.898 (Fig. 5). To select the optimal configuration, the Delong test was used to evaluate the diagnostic performance of the two machine learning models, and no significant difference in the AUC values for the models was detected (AUC for the KNN-based model = 0.849 vs. AUC for the random forest-based model = 0.898, *P* = 0.6964). Therefore, we selected the model constructed using the KNN method combined with T2WI and T1CE sequence features as the radiomics model with the best stability.

### Validation of radiomics model

The AUC value of the radiomic model constructed using the KNN method was 0.849 (0.804–0.887) with a sensitivity of 0.784 and specificity of 0.805. For this model, the calibration curve showed good consistency with the ideal curve. In addition, we also compared the diagnostic performance of the developed model with that of traditional models constructed using logistic regression for all combinations of features. The Delong test showed no significant difference between the KNN-based model and the traditional logistic regression model constructed using T1WI + DWI + T1CE and T2WI + T1WI + DWI + T1CE data (*P* > 0.05), but statistical differences were detected among other models (*P* < 0.05). Moreover, the developed radiomics model showed the highest diagnostic performance (Table 2).

**Table 2:** Diagnostic performance the traditional logistic regression models based on different MRI sequence combinations compared with the developed KNN-based model.

Models	Sample dataset (n = 308)		
	AUC (95% CI)	Sensitivity	Specificity
T2WI + T1WI	0.770 (0.719–0.816)	0.824	0.627
T2WI + DWI	0.722 (0.669–0.772)	0.628	0.708
T2WI + T1CE	0.730 (0.677–0.779)	0.824	0.58
T1WI + DWI	0.774 (0.723–0.819)	0.745	0.708
T1WI + T1CE	0.754 (0.702–0.801)	0.765	0.677
DWI + T1CE	0.694 (0.639–0.745)	0.49	0.844
T2WI + T1WI + DWI	0.768 (0.717–0.814)	0.706	0.751
T2WI + T1WI + T1CE	0.772 (0.721–0.818)	0.843	0.658
T2WI + DWI + T1CE	0.773 (0.722–0.819)	0.765	0.669
T1WI + DWI + T1CE	0.820 (0.773–0.862)	0.882	0.712
T2WI + T1WI + DWI + T1CE	0.840 (0.795–0.88)	0.804	0.782
KNN signature	0.849 (0.804–0.887)	0.784	0.805

**Figure 6:**
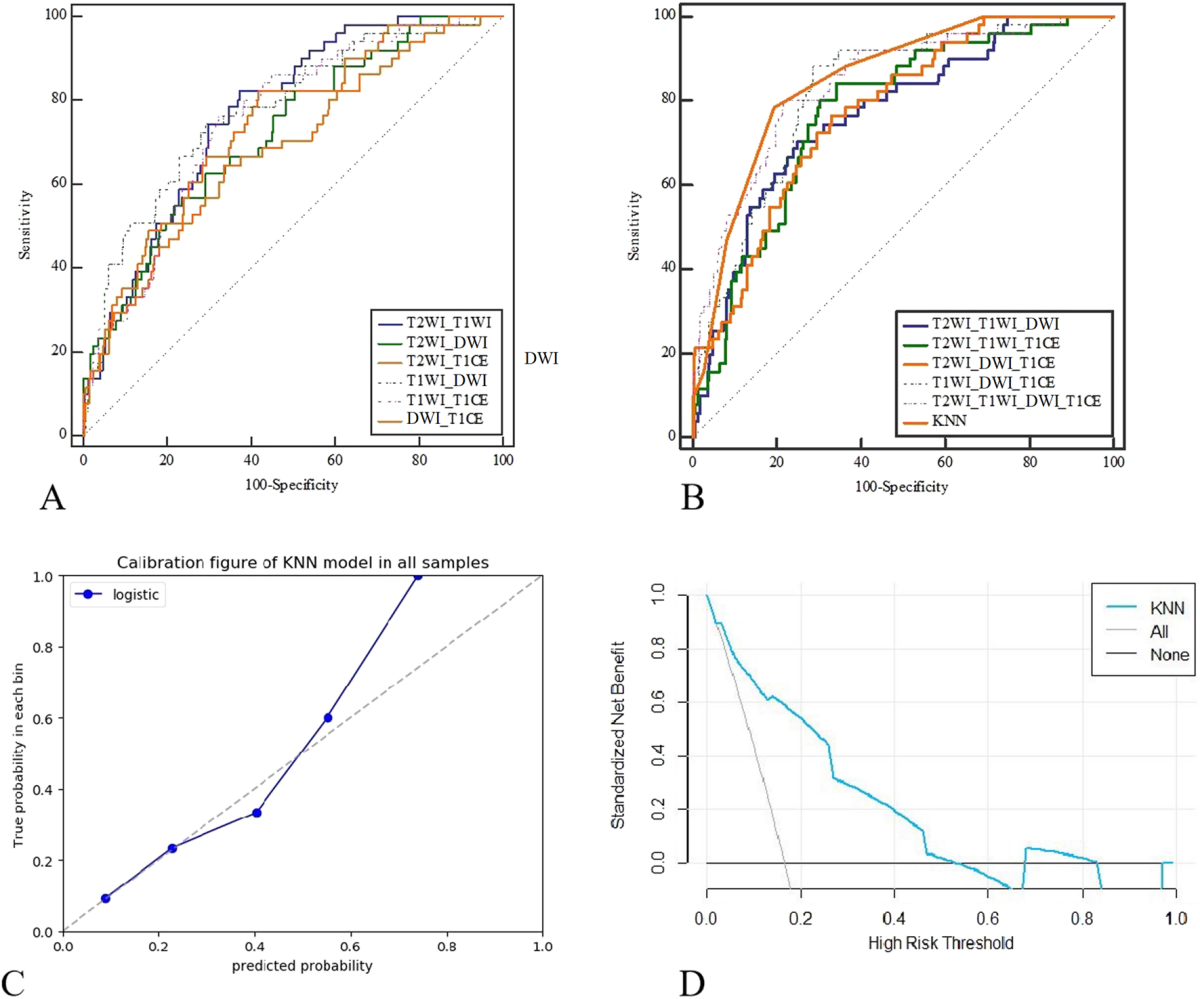
ROC curve analysis of different models for the preoperative prediction of MSI in RC patients. (**a**) Diagnostic performance of traditional models constructed by combining two sequence types. (**b**) Diagnostic performance of traditional models combining more than two sequence types and the developed KNN-based model. (**c**) Correction curve for the KNN-based model. The dotted line represents ideal prediction performance, and the solid line represents the actual prediction performance. (**d**) Static benefit analysis of the developed model.

Finally, in our evaluation of the clinical performance of the radiomics model, ROC curve analysis showed that the optimal cut-off value corresponding to the Youden index threshold was 0.2 for distinguishing low probability and high probability groups. Notably, the number of MSI patients differed between the low probability and high probability groups, supporting the clinical applicability of the model (Table 3, Fig. 6). The number of pathologically confirmed cases with MSI in the high probability group using the model classification was indeed higher than that in the low probability group.

**Table 3:** Clinical classification performance of the developed machine learning-based radiomics model.

Group		Number of cases (n,%)	Pathological result		*P*
			MSI (n,%)	MSS (n,%)	
All patients	Low-risk	218 (70.78)	11 (81.88)	207 (18.12)	< 0.001
(N = 308)	High-risk	90 (29.22)	40 (25.12)	50 (74.88)

## Discussion

Radiomics can capture microscopic information from images that cannot be detected by visual inspection. This microscopy information may reflect the biological heterogeneity of tissues and provide accurate information regarding pathological changes, and thus, radiomics data can be valuable for the differential diagnosis of tumors, evaluation of pathological grading, and prognosis prediction^14^. The results of the present study showed that the selected radiomics features based on different sequences could well identify the MSI status of RC patients, especially when the radiomics features of T2WI and T1CE images were combined, with this model exhibiting the best performance for prediction of MSI status. In addition, radiomics models based on KNN using two sequences showed better predictive performance than traditional logistic regression models using four sequences, further demonstrating the advantages of machine learning in medical imaging-based prediction models.

MSI status is a key determining factor for the prognosis and treatment of RC^15–17^. In recent years, radiomics has become a promising tool for the noninvasive prediction of MSI status in RC^18,19^. The present study describes the development and validation of an optimal radiomics model for preoperative prediction of MSI status in RC patients. In our analyses, we compared the performance of models constructed using five machine learning algorithms, including the KNN, SVM, Bayes, decision tree, and random forest methods. These machine learning algorithms individually were combined with 11 optimal combinations of imaging features to construct radiomics models. The results showed that the KNN method was superior to the other four machine learning algorithms in terms of the AUC, and stability of the prediction radiomics model. This finding was consistent with the results reported by Granata et al. in their study of liver mucinous colorectal metastases^20^. In another study of RAS mutation prediction based on contrast-enhanced MRI in cases of colorectal cancer with liver metastasis^21^, the KNN method also showed great accuracy, sensitivity, and specificity (training set: accuracy = 76.9%, sensitivity = 90.0%, and specificity = 67.8%; verification set: accuracy = 87.5%, sensitivity = 91.7%, and specificity = 83.3%). The KNN algorithm is simple and easy to apply, with few assumptions regarding data distribution, and it is suitable for various types of data, including medical imaging data. Compared with other machine learning algorithms, such as the SVM and decision tree, overfitting is less likely to occur with the KNN algorithm^22^.

In the present study, the combination of T2WI and T1CE sequences showed the highest prediction performance among all combinations of sequences. This was consistent with the results reported by Jiang et al.^23^, who found that a radiomics model using T2WI and T1CE sequences provided good performance for detecting MRI-invisible early-stage endometrial cancer and myometrial invasions. Hu et al.^24^ combined T1CE and T2WI radiomics analysis of cervical mucosa to predict early cervical cancer not visible by MRI. Previous research also indicated that T2WI and T1CE sequences may be able to predict MSI status in RC patients, possibly due to the high contrast between the rectal wall and surrounding tissues on T2WI sequences and the enhancement of rectal blood vessels and lymph nodes on T1CE sequences. The results of the present study also indicate that the combination of T2WI and T1CE sequences can better identify and characterize tumor lesions and surrounding tissues than other sequence combinations, as this combination showed the best performance for predicting MSI status.

In this study, the radiomics model constructed by using the KNN method with T2WI and T1CE sequence features showed similar diagnostic performance compared with the traditional model constructed by logistic regression. The performance of the developed model even appeared to be slightly better, but the difference was not found to be statistically significant. The findings of this study indicate that radiomics features based on MRI might also be a feasible method, which may provide a novel tool for clinical practice. Radiomics data have been used to predict pathological statuses in the context of various diseases, including the expression level of human epidermal growth factor receptor 2^25,26^ and lymph node metastasis^27,28^ in patients with breast cancer. Therefore, the present study showing the potential for predicting MSI status further expands the range of radiomics applications.

The present study does have several limitations. First, the sample size was small, and all patient data were collected from a single center. Further studies analyzing larger sample sizes collected from multiple centers are needed to validate our results. Second, we only used MRI data to construct our radiomics model for MSI status prediction in RC patients, and future studies should examine whether the accuracy of the prediction model can be further improved by including data from additional imaging methods such as computed tomography (CT) and positron emission tomography (PET).

## Conclusions

In conclusion, we developed and validated a radiomics model based on machine learning and an optimal combination of MRI sequence features for the preoperative prediction of MSI status in RC patients. Our analyses indicated that the KNN method was the best machine learning algorithm for MRI radiomics-based prediction of MSI status, and T2WI and T1CE data offered the best sequence combination. Thus, the present study provides a new method for predicting preoperative MSI status in RC patients and may be applied to obtain beneficial guidance for personalized treatment decisions.

### Supplementary Information

Supplementary Information.

## Data Availability

The datasets generated during and/or analysed during the current study are available from the corresponding author on reasonable request.
