# Supplementary material for: A combinatorial MRI sequence-based radiomics model for preoperative prediction of microsatellite instability status in rectal cancer
Source: Sci Rep. 2024 May 23;14:11760. doi: 10.1038/s41598-024-62584-0 (PMC11116457; doi:10.1038/s41598-024-62584-0)
Supplement: Supplementary file 1 — Supplementary Information. [file 41598_2024_62584_MOESM1_ESM.pdf]

Supplementary Materials

1. Clinical and radiological variables

Clinical variables include age, gender andcarcinoembryonic antigen (CEA). We considered a CEA level greater than 5 ng/mL as abnormal.

Radiological variables include MRI-based extramural vascular invasion (mrEMVI) status, circumferential resection margin (CRM) status, distance (DIS), radiological tumor (T) stage and lymph node (N) stage. Positive mrEMVI is defined as (a) the presence of tumor signal intensity within a vascular structure, (b) expanded vessels, and (c) tumoral expansion through and beyond the vessel wall, disrupting the vessel border. Positive CRM is defined as the condition when the tumor, lymph node, EMVI, or tumoral deposits are within 1 mm of the meso-rectal fascia. DIS is defined as the distance from the end of the convex edge of the tumor to the edge of the anus. Radiological T staging are divided into T_{1-2} and T_{3-4}. Lymph node metastasis is defined as (a) the short-axis diameter of suspicious lymph nodes ≥9 mm; (b) short-axis diameter between 5 and 8 mm, with more than two morphologically suspicious features (including irregular border, round shape, and heterogeneous signal); (c) short-axis diameter < 5 mm, with irregular border, round shape, and heterogeneous signal; and (d) all mucinous lymph nodes which showed T_2WI high signal (any size). These standards are widely accepted and applied in routine clinical practice[1, 2].

2. MRI scan

All patients underwent placental MRI using 3.0T MR image systems, either with (Skyra; Siemens Healthineers) equipped with an 8-channel phased-array coil in supine position. The MRI sequences including (1) Sagittal T2WI, (2) Axial T2 blade TSE, (3) Axial resolve DWI, (4) Enhanced T1WI. The intravenous injection of Gadolinium contrast agent (Magnevist, Bayer, Germany) were performed for each patient. The detailed parameters for each sequence were illustrated in Table S1.

Table S1. MRI parameters of each sequence

Scanner	Sequence	Orientation	TR (ms)	TE (ms)	FOV (mm^2)	Thickness (mm)	Interslice gap (mm)	Matrix
SIEMENS 3.0T (Skyra)	T_2WI	Sagittal	6060	90	180´ 180	3	0.6	320´ 224
	T_2WI	Axial	4790	134	200´ 200	3	0.6	384´ 451
	T_1WI	Axial	662	9.6	180´ 180	3	0.6	320´ 224
	DWI	Axial	7330	56.0	200´ 200	3	0.8	112´ 100
	T_1CE	Axial	616	9.6	180´ 180	3	0.6	320´ 224

Note. TR, repetition time; TE, echo time; FOV, field of view.

3. Image preprocessing and segmentation

Image preprocessing including resample, intensity normalization and gray-level discretization were performed with AK software (Analysis Kit, GE Healthcare). To be more specific, image preprocessing was performed by resampling the images with a resolution of 1×1×1 mm^3 through the linear interpolation method and by discretizing and normalizing the image gray level to order 32. Extracted texture features were standardized, which removed the unit limits of the data of each feature and converted it into a dimensionless pure value. This allowed the indexes of different units or orders to be compared and weighted. We used a z-score normalization to make the image intensities fit a standard normal distribution with $\mu=0$ and $\sigma=1$, where μ is the mean value of the images, and σ is the standard deviation. The normalized values (also called z-scores) of the image intensities (x) were calculated as follows:

$$z=\frac{x-\mu}{\sigma}$$

Image segmentation program is as follows. Firstly, SPM12 software was used to rigorously register the images of T_2WI, T_1WI, DWI, and T_1CE sequences in order to reduce the potential influence of the parameters of a scanning scheme. After that, the standardized T_2WI images were imported into the ITK software to manually segment the entire rectal tumor layer by layer and to determine the volume of interest (VOI). Since the four sequences have been rigorously registered, tumor VOI obtained from T_2WI can be applied directly to other sequences. All cases undertook the same VOI segmentation method.

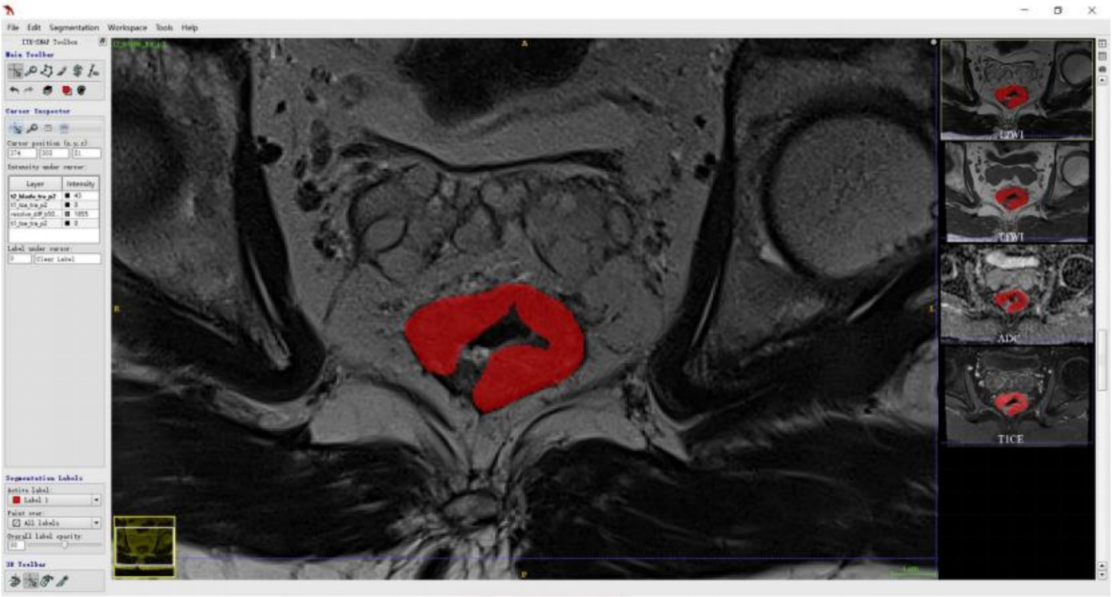

Figure S1. Representative manual segmentation of the entire rectal tumor in the T_2WI, T_1WI, DWI, and T_1CE using ITK software.

Table S2. The information of radiomics features

Feature Groups (N)	Feature names	Feature Groups (N)	Feature names
First-order features (N = 18)	firstorder_10Percentile	GLSZM texture features (N = 16)	glszm_GrayLevelNonUniformity
	firstorder_90Percentile		glszm_GrayLevelNonUniformityNormalized
	firstorder_Energy		glszm_GrayLevelVariance
	firstorder_Entropy		glszm_HighGrayLevelZoneEmphasis
	firstorder_InterquartileRange		glszm_LargeAreaEmphasis
	firstorder_Kurtosis		glszm_LargeAreaHighGrayLevelEmphasis
	firstorder_Maximum		glszm_LargeAreaLowGrayLevelEmphasis
	firstorder_MeanAbsoluteDeviation		glszm_LowGrayLevelZoneEmphasis
	firstorder_Mean		glszm_SizeZoneNonUniformity
	firstorder_Median		glszm_SizeZoneNonUniformityNormalized
	firstorder_Minimum		glszm_SmallAreaEmphasis
	firstorder_Range		glszm_SmallAreaHighGrayLevelEmphasis
	firstorder_RobustMeanAb		glszm_SmallAreaLow

	soluteDeviation		GrayLevelEmphasis
	firstorder_RootMeanSquared		glszm_ZoneEntropy
	firstorder_Skewness		glszm_ZonePercentage
	firstorder_TotalEnergy		glszm_ZoneVariance
	firstorder_Uniformity		
GLRLM texture features (N = 16)	glrlm_GrayLevelNonUniformity	GLCM texture features (N = 24)	glcm_Autocorrelation
	glrlm_GrayLevelNonUniformityNormalized		glcm_ClusterProminence
	glrlm_GrayLevelVariance		glcm_ClusterShade
	glrlm_HighGrayLevelRunEmphasis		glcm_ClusterTendency
	glrlm_LongRunEmphasis		glcm_Contrast
	glrlm_LongRunHighGrayLevelEmphasis		glcm_Correlation
	glrlm_LongRunLowGrayLevelEmphasis		glcm_DifferenceAverage
	glrlm_LowGrayLevelRunEmphasis		glcm_DifferenceEntropy
	glrlm_RunEntropy		glcm_DifferenceVariance
	glrlm_RunLengthNonUniformity		glcm_Id
	glrlm_RunLengthNonUniformityNormalized		glcm_Idm
	glrlm_RunPercentage_T2		glcm_Idmn
	glrlm_RunVariance		glcm_Idn
	glrlm_ShortRunEmphasis		glcm_Imc1

	glrlm_ShortRunHighGrayLevelEmphasis<br>glrlm_ShortRunLowGrayLevelEmphasis		glcm_Imc2<br>glcm_InverseVariance<br>glcm_JointAverage<br>glcm_JointEnergy<br>glcm_JointEntropy<br>glcm_MCC<br>glcm_MaximumProbability<br>glcm_SumAverage<br>glcm_SumEntropy<br>glcm_SumSquares
GLDM texture features<br>(N = 14)	gldm_DependenceEntropy<br>gldm_DependenceNonUniformity<br>gldm_DependenceNonUniformityNormalized<br>gldm_DependenceVariance<br>gldm_GrayLevelNonUniformity<br>gldm_GrayLevelVariance<br>gldm_HighGrayLevelEmphasis<br>gldm_LargeDependenceEmphasis<br>gldm_LargeDependenceH	NGTDM texture features<br>(N = 5)	ngtdm_Busyness<br>ngtdm_Coarseness<br>ngtdm_Complexity<br>ngtdm_Contrast<br>ngtdm_Strength

	ighGrayLevelEmphasis<br>gldm_LargeDependenceL<br>owGrayLevelEmphasis<br>gldm_LowGrayLevelEmp<br>hasis<br>gldm_SmallDependenceE<br>mphasis<br>gldm_SmallDependenceH<br>ighGrayLevelEmphasis<br>gldm_SmallDependenceL<br>owGrayLevelEmphasis
Los features (N = 186)	Log-sigma-1.0， 2.0_* (N =186)

Note: GLCM, Gray-level co-occurrence matrices; GLRLM, Gray-level run length matrix; GLSZM, Gray-level size zone matrix; GLDM, Gray-level dependence matrix. *The abbreviated representation of feature types

Table S2. The detailed information of remaining radiomics features

Sequence	Feature	Category
T_2WI (n=11)	log-sigma-2-0-mm-3D_firstorder_Kurtosis_T2	Firstorder
	original_firstorder_Skewness_T2	Firstorder
	wavelet-LLH_firstorder_InterquartileRange_T2	Firstorder
	wavelet-LHL_glszm_GrayLevelNonUniformity_T2	GLSZM
	wavelet-LHL_glszm_SmallAreaLowGrayLevelEmphasis_T2	GLSZM
	wavelet-HLL_glcm_ClusterShade_T2	GLCM
	wavelet-HLL_gldm_LargeDependenceHighGrayLevelEmphasis_T2	GLDM
	wavelet-HLH_glcm_MCC_T2	GLCM
	wavelet-HHL_gldm_DependenceNonUniformityNormalized_T2	GLDM
	wavelet-HHL_glrlm_HighGrayLevelRunEmphasis_T2	GLRLM
	wavelet-LLL_glcm_Correlation_T2	GLCM

T_1WI (n=11)	wavelet-LHL_glcm_Imc1_T1	GLCM
	wavelet-LLL_glrlm_RunLengthNonUniformity_T1	GLRLM
	wavelet-LHH_gldm_LargeDependenceHighGrayLevelEmphasis_T1	GLDM
	log-sigma-3-0-mm-3D_glszm_LowGrayLevelZoneEmphasis_T1	GLSZM
	original_gldm_DependenceVariance_T1	GLDM
	wavelet-LLH_glcm_JointEntropy_T1	GLCM
	log-sigma-2-0-mm-3D_firstorder_Skewness_T1	Firstorder
	wavelet-HLL_glcm_MCC_T1	GLCM
	wavelet-HHL_glszm_SizeZoneNonUniformityNormalized_T1	GLSZM
	wavelet-HHH_glcm_JointAverage_T1	GLCM
	log-sigma-2-0-mm-3D_glszm_GrayLevelNonUniformity_T1	GLSZM
DWI (n=10)	wavelet-LLH_firstorder_Kurtosis_DWI	Firstorder
	wavelet-LLH_glcm_MaximumProbability_DWI	GLCM
	wavelet-LHL_firstorder_Minimum_DWI	Firstorder
	wavelet-LHL_gldm_DependenceEntropy_DWI	GLDM
	wavelet-HLL_glcm_Idn_DWI	GLCM
	wavelet-HLH_glcm_MaximumProbability_DWI	GLCM
	wavelet-HHL_glrlm_HighGrayLevelRunEmphasis_DWI	GLRLM
	wavelet-HHH_firstorder_Skewness_DWI	Firstorder
	wavelet-HHH_firstorder_TotalEnergy_DWI	Firstorder
	wavelet-LLL_gldm_DependenceVariance_DWI	GLDM
T_1CE (n=15)	log-sigma-2-0-mm-3D_glcm_ClusterShade_T1CE	GLCM
	wavelet-HLL_glszm_ZonePercentage_T1CE	GLSZM
	wavelet-HHH_gldm_DependenceVariance_T1CE	GLDM
	original_glrlm_ShortRunLowGrayLevelEmphasis_T1CE	GLRLM
	wavelet-HHH_glszm_SmallAreaLowGrayLevelEmphasis_T1CE	GLSZM
	wavelet-LHH_glcm_Imc2_T1CE	GLCM
	original_glszm_ZoneVariance_T1CE	GLSZM
	wavelet-LLL_glszm_ZonePercentage_T1CE	GLSZM
	original_ngtdm_Strength_T1CE	Ngtdm
	wavelet-HHH_glszm_SmallAreaHighGrayLevelEmphasis_T1CE	GLSZM
	wavelet-LLH_gldm_DependenceVariance_T1CE	GLDM
	wavelet-LLL_firstorder_Kurtosis_T1CE	Firstorder
	original_glszm_SmallAreaHighGrayLevelEmphasis_T1CE	GLSZM
	wavelet-LLL_glrlm_RunLengthNonUniformity_T1CE	GLRLM
	wavelet-HLH_glcm_SumEntropy_T1CE	GLCM

Note. Rad-score of the fusion model can be calculated by intercept and their respective coefficients. intercept = -1.515.

Table S3. Multi-factor logistic regression analysis based on different sequences optimal feature combinations

Combinated Sequences	Feature names	OR（95%CI）	*P*-value
T2WI+T1WI	wavelet-LHL_glcm_Imc1_T1	0.643（0.467, 0.885）	0.007
	wavelet-HLL_glcm_MCC_T1	1.418（1.027, 1.957）	0.034
	wavelet-LLH_firstorder_InterquartileRange_T2	1.712（1.256, 2.334）	0.001
	wavelet-LHL_glszm_GrayLevelNonUniformity_T2	1.439（1.049, 1.973）	0.024
	wavelet-HLL_gldm_LargeDependenceHighGrayLevelEmphasis_T2	0.510（0.344, 0.756）	0.001
	wavelet-LLL_glcm_Correlation_T2	1.543（1.075, 2.214）	0.019
T1WI+DWI	wavelet-LHL_glcm_Imc1_T1	0.659（0.481, 0.903）	0.009
	wavelet-LLL_glrlm_RunLengthNonUniformity_T1	1.580（1.135, 2.200）	0.007
	wavelet-LHH_gldm_LargeDependenceHighGrayLevelEmphasis_T1	0.646（0.447, 0.933）	0.02
	log-sigma-2-0-mm-3D_firstorder_Skewness_T1	0.661（0.474, 0.921）	0.014
	wavelet-LHL_gldm_DependenceEntropy_DWI	0.594（0.412, 0.856)	0.005
	wavelet-HLH_glcm_MaximumProbability_DWI	0.590（0.406, 0.857）	0.006
	wavelet-HHH_firstorder_Skewness_DWI	0.663（0.469, 0.937）	0.02
	wavelet-LLL_gldm_DependenceVariance_DWI	1.603（1.139, 2.257）	0.007
T1WI+T1CE	wavelet-LHL_glcm_Imc1_T1	0.637（0.463, 0.879）	0.006
	original_gldm_DependenceVariance_T1	1.739（1.189, 2.545）	0.004
	wavelet-HLL_glszm_ZonePercentage_T1CE	1.550（1.134, 2.118）	0.006
	wavelet-HHH_gldm_DependenceVariance_T1CE	0.466（0.300, 0.722）	0.001
	wavelet-HHH_glszm_SmallAreaLowGrayLevelEmphasis_T1CE	1.402（1.028, 1.913）	0.033

	wavelet-LLL_glszm_ZonePercentage_T1CE	0.668（0.461，0.970）	0.034
	wavelet-LLH_firstorder_InterquartileRange_T2	1.737（1.272，2.371）	0.001
**T2WI+DWI**	wavelet-HLL_gldm_LargeDependenceHighGrayLevelEmphasis_T2	0.644（0.443，0.936）	0.021
	wavelet-LHL_gldm_DependenceEntropy_DWI	0.626（0.454，0.864）	0.004
	wavelet-LLL_gldm_DependenceVariance_DWI	1.560（1.137，2.141）	0.006
	wavelet-LLH_firstorder_InterquartileRange_T2	1.657（1.224，2.244）	0.001
**T2WI+T1CE**	wavelet-LHL_glszm_GrayLevelNonUniformity_T2	1.403（1.041，1.892）	0.026
	wavelet-HLL_gldm_LargeDependenceHighGrayLevelEmphasis_T2	0.476（0.316，0.716）	<0.001
	wavelet-LLL_glszm_ZonePercentage_T1CE	0.572（0.388，0.842）	0.005
	wavelet-LHL_gldm_DependenceEntropy_DWI	0.642（0.470，0.877）	0.005
**DWI+T1CE**	wavelet-LLL_gldm_DependenceVariance_DWI	1.476（1.064，2.049）	0.02
	wavelet-HHH_gldm_DependenceVariance_T1CE	0.622（0.438，0.884）	0.008
	wavelet-HHH_glszm_SmallAreaLowGrayLevelEmphasis_T1CE	1.457（1.081，1.963）	0.013
	wavelet-LHL_glcm_Imc1_T1	0.666（0.488，0.909）	0.01
	log-sigma-2-0-mm-3D_firstorder_Skewness_T1	1.986（1.421，2.776）	<0.001
	wavelet-LHL_glszm_GrayLevelNonUniformity_T2	1.649（1.205，2.255）	0.002
**T2WI+T1WI+DWI**	wavelet-HLL_gldm_LargeDependenceHighGrayLevelEmphasis_T2	0.605（0.411，0.891）	0.011
	wavelet-LHL_gldm_DependenceEntropy_DWI	0.666（0.487，0.911）	0.011
	wavelet-HLH_glcm_MaximumProbability_DWI	0.652（0.481，0.882）	0.006
	wavelet-LLL_gldm_DependenceVariance_DWI	1.789（1.322，2.421）	<0.001
**T2WI+T1WI+T1CE**	wavelet-LHL_glcm_Imc1_T1	0.635（0.462，0.873）	0.005
	original_gldm_DependenceVariance_T1	1.693（1.157，2.478）	0.007

	wavelet-HLL_glcm_MCC_T1	1.435（1.044，1.972）	0.026
	wavelet-LLH_firstorder_InterquartileRange_T2	1.409（1.068，1.859）	0.015
	wavelet-LHL_glszm_GrayLevelNonUniformity_T2	1.524（1.135，2.047）	0.005
	wavelet-HLL_glszm_ZonePercentage_T1CE	1.425（1.058，1.918）	0.02
	wavelet-HHH_gldm_DependenceVariance_T1CE	0.449（0.305，0.662）	<0.001
**T2WI+T1CE+DWI**	wavelet-LLH_firstorder_InterquartileRange_T2	1.841（1.318，2.571）	<0.001
	wavelet-LHL_glszm_GrayLevelNonUniformity_T2	1.568（1.136，2.165）	0.006
	wavelet-HLL_gldm_LargeDependenceHighGrayLevelEmphasis_T2	0.450（0.285，0.711）	0.001
	wavelet-LLL_glszm_ZonePercentage_T1CE	0.596（0.396，0.897）	0.013
	wavelet-LHL_gldm_DependenceEntropy_DWI	0.594（0.423，0.833）	0.003
	wavelet-HLH_glcm_MaximumProbability_DWI	0.598（0.405，0.885）	0.01
	wavelet-LLL_gldm_DependenceVariance_DWI	1.548（1.103，2.174）	0.012
**T1WI+DWI+T1CE**	wavelet-HLL_glszm_ZonePercentage_T1CE	1.676（1.143，2.459）	0.008
	wavelet-HHH_gldm_DependenceVariance_T1CE	0.440（0.269，0.718）	0.001
	wavelet-HHH_glszm_SmallAreaLowGrayLevelEmphasis_T1CE	1.513（1.077，2.125）	0.017
	wavelet-HLH_glcm_SumEntropy_T1CE	1.521（1.035，2.234）	0.033
	wavelet-LHL_gldm_DependenceEntropy_DWI	0.590（0.407，0.855）	0.005
	wavelet-HLH_glcm_MaximumProbability_DWI	0.588（0.389，0.888）	0.012
	wavelet-HHH_firstorder_Skewness_DWI	0.679（0.479，0.961）	0.029
	wavelet-LHL_glcm_Imc1_T1	0.581（0.412，0.820）	0.002
	wavelet-LLL_glrlm_RunLengthNonUniformity_T1	1.810（1.249，2.623）	0.002
	original_gldm_DependenceVariance_T1	1.429（1.012，2.017）	0.043

T2WI+T1WI+DWI+T1CE	original_gldm_DependenceVariance_T1	1.917（1.248，2.945）	0.003
	wavelet-HLL_glszm_ZonePercentage_T1CE	1.784（1.199，2.656）	0.004
	wavelet-HHH_gldm_DependenceVariance_T1CE	0.375（0.220，0.639）	<0.001
	wavelet-HHH_glszm_SmallAreaLowGrayLevelEmphasis_T1CE	1.565（1.092，2.242）	0.015
	wavelet-HLH_glcm_SumEntropy_T1CE	1.649（1.095，2.481）	0.017
	wavelet-LHL_gldm_DependenceEntropy_DWI	0.585（0.395，0.866）	0.007
	wavelet-HLH_glcm_MaximumProbability_DWI	0.502（0.319，0.790）	0.003
	wavelet-HHH_firstorder_Skewness_DWI	0.659（0.459,0.948）	0.025
	wavelet-LLL_gldm_DependenceVariance_DWI	1.470（1.007，2.145）	0.046
	wavelet-LHL_glcm_Imc1_T1	0.627（0.437，0.900）	0.011
	wavelet-LLL_glrlm_RunLengthNonUniformity_T1	1.827（1.242，2.687）	0.002
	original_gldm_DependenceVariance_T1	1.808（1.130，2.892）	0.014
	log-sigma-2-0-mm-3D_firstorder_Skewness_T1	0.631（0.429，0.928）	0.019
	wavelet-HLL_glcm_MCC_T1	1.449（1.019，2.059）	0.039
	wavelet-HLL_glcm_ClusterShade_T2	1.583（1.086，2.306）	0.017

Reference

1 Horvat N, Carlos Tavares Rocha C, Clemente Oliveira B, Petkovska I, Gollub MJ (2019) MRI of Rectal Cancer: Tumor Staging, Imaging Techniques, and Management. Radiographics 39:367-387

2 Nougaret S, Jhaveri K, Kassam Z, Lall C, Kim DH (2019) Rectal cancer MR staging: pearls and pitfalls at baseline examination. Abdom Radiol (NY) 44:3536-3548
